# Early Oral Feeding with Vascular Resection among Patients Undergoing Pancreatoduodenectomy in a Tertiary Care Hospital: A Descriptive Cross-sectional Study

**DOI:** 10.31729/jnma.7272

**Published:** 2022-02-28

**Authors:** Sujan Regmee, Yugal Limbu, Anuj Parajuli, Roshan Ghimire, Dhiresh Kumar Maharjan, Suman Shrestha, Prabin Bikram Thapa

**Affiliations:** 1 Department of Surgery, Kathmandu Medical College and Teaching Hospital, Sinamangal, Kathmandu, Nepal

**Keywords:** *pancreatoduodenectomy*, *portal vein*, *superior mesenteric artery*, *vascular surgery*

## Abstract

**Introduction:**

Pancreatoduodenectomy with vascular resection is performed in locally advanced periampullary malignancies. In our practice, early oral feeding is initiated in patients undergoing pancreatoduodenectomy. This study aims to find the prevalence of early oral feeding with vascular resection among patients undergoing pancreatoduodenectomy.

**Methods:**

This was a descriptive cross-sectional study conducted among hospital records of 152 patients who underwent pancreatoduodenectomy in the department of surgery of a tertiary care hospital from 2016 to 2020. Ethical approval was taken from the Institutional Review Committee (Reference number: 0812202102). Convenience sampling was done. Patients clinical and sociodemographic data were collected and analyzed using Statistical Package for the Social Sciences version 20. Point estimate at 95% Confidence Interval was calculated along with frequency, percentage, mean, and median.

**Results:**

Among 152 patients undergoing pancreatoduodenectomy, early oral feeding with vascular resection was done in 17 (11.18%) (6.17-16.19 at 95% Confidence Interval). Portal vein and superior mesenteric artery were resected in one (5.88%) and hepatic artery in one (5.88%) patient. Type I, III and IV reconstruction was done in nine (52.9%), five (29.41%) and one (5.88%) respectively. Clinically relevant delayed gastric emptying and postoperative pancreatic fistula were seen in two (11.7%). Complication of Clavien-Dindo Grade III or higher was seen in one (5.88%) patient. One (5.88%) mortality was noted.

**Conclusions:**

The prevalence of early oral feeding with vascular resection among patients undergoing pancreatoduodenectomy was similar to other studies done in similar settings. Early enteral feeding is well tolerated in patients undergoing pancreatoduodenectomy with vascular resection.

## INTRODUCTION

Pancreatoduodenectomy with vascular resection is done in locally advanced periampullary tumors to attain R_0_ resection. Amongst the many feeding route early oral feeding is considered to be the best route.^1,2^ Surgeons are reluctant to start early oral feeding in pancreatoduodenectomy as it promotes pancreatic secretion and increases the chances of post-operative pancreatic failure or delayed gastric emptying (DGE).^3^

Factors reported to be associated with intolerance of oral feeding are dysrhythmia of the stomach, ischemia of the pyloric muscles, gastroduodenal neural connection disruption, and ligation of the right gastric artery.^4^ Pre-operative association of diabetes mellitus, cholangitis, and previous abdominal surgeries are also associated with the condition.^5^

This study aims to find the prevalence of early oral feeding with vascular resection among patients undergoing pancreatoduodenectomy.

## METHODS

This was a descriptive cross-sectional study, conducted at the Department of Surgery in Kathmandu Medical College and Teaching Hospital, Sinamangal, Kathmandu. Ethical approval was taken from the Institutional Review Committee (Reference number: 0812202102). Data was collected retrospectively from the department record of patients who underwent pancreatoduodenectomy, from 2016 to 2020. All patients undergoing pancreatoduodenectomy without pylorus preservation with pancreaticojejunostomy were included in the study. Convenience sampling was done and the sample size was calculated using the formula,

n = Z^2^ × p × q / e^2^

  = (1.96)^2^ × 0.5 × 0.5 / (0.08)^2^

  = 151

Where,

n = minimum required sample size
Z = 1.96 at 95% Confidence Interval (CI)
p = prevalence taken as 50% for maximum sample size
q = 1-p
e = margin of error, 8%

The calculated sample size was 151. However, we included 152 patients in the study. All surgeries were performed by laparotomy with a chevron incision, and a standard pancreatoduodenectomy was performed without pylorus preservation. Standard lymphadenectomy was performed in all the cases included in the analysis. Extended dissection of the celiac lymph nodes (now known as triangle operation) was performed in 5 patients. End to side anti-colic pancreaticojejunostomy was performed using the standard Blumgart reconstruction technique. End to side hepaticojejunostomy and anti-colic end to side gastrojejunostomy was performed. Naso gastric tube was taken out at the end of the surgery. Intraoperative and post-operative octreotide was used routinely in all the patients. All patients were shifted to the intensive care unit at the end of the procedure.

With the intent of early oral feeding patients were planned to start on sips of clear liquid from the day of surgery six hours after extubation or on the first postoperative day if elective intubation is continued. A clear liquid diet was allowed on postoperative day two and a soft diet was started from the third postoperative day. Intravenous and epidural analgesia was used in all the patients.

Tolerance of oral liquid diet, time of appearance of bowel sound, time of passage of flatus/stool, the incidence of vomiting, time of reinsertion of NG tube (if required), post-op days of IV fluid, tolerance of normal diet, and discharge were measured. Tolerance to oral feeding was noted in terms of the development of symptoms like nausea and vomiting, abdominal bloating, crampy abdominal pain, and episodes of diarrhea. The occurance of Clinically relevant Delayed Gastric Emptying (Cr-DGE), Clinically relevant Postoperative Pancreatic Fistula (Cr-POPF) as defined by the International Study Group of Pancreatic Surgery (ISGPS),^6^ the complication of Clavien-Dindo Grade III or higher were noted.

Data was entered and analyzed using Statistical Package for the Social Sciences version 20. Point estimate at 95% Confidence Interval was calculated along with descriptive statistics like frequency, percentages, mean and median.

## RESULTS

Among 152 patients undergoing pancreatoduodenectomy, early oral feeding with vascular resection was done in 17 (11.18%) (6.17-16.19 at 95% Confidence Interval). Of the 17 patients who had early oral feeding, the mean age was 65.2±10.5 years. Eight (47%) were males and nine (53%) were females. About six (35.3%) patients had preoperatively diagnosed diabetes mellitus. Five (29.4%) patients required preoperative biliary drainage and seven (41.1%) patients had hypoalbuminemia. About five (29.4%) patients underwent triangle surgery (Table 1).

**Table 1:** Patient demographics and tumor location (n = 17).

Demographic profile	n (%)
**Gender**	
Male	8 (47)
Female	9 (53)
**Comorbidities**	
Presence of Diabetes mellitus	6 (35.3)
Preoperative biliary drainage	5 (29.4)
Preoperative hypoalbuminemia	7 (41.1)
**Location of Tumor**	
Head of pancreas	12 (70.5)
Uncinate process of pancreas	4 (23.5)
Distal CBD	1 (5.8)
Ampullary	-
Duodenal	-
**Extent of lymph node dissection**	
Triangle operation	5 (29.5)

The average operative time for pancreaticoduodenectomy with vascular resection was 510±120 minutes. The average estimated blood loss was 1600±1500ml. On average four units of blood were transfused.

**Table 2:** Intra-operative characteristics (n= 17).

Intraoperative factors	Mean
Operative time (min)	510±120
Intraoperative blood loss (ml)	1600±1500
Perioperative blood transfusion (units)	4±5

The median duration of intensive care stay was 4 days and the median hospital stay was 9 days. One patient required reoperation for postoperative hemorrhage (Table 3).

**Table 3:** Postoperative characteristics.

Postoperative factors	Median duration
Median ICU stay	4
Median hospital stay	9
Re-operation	1

The portal vein, superior mesenteric artery, superior mesenteric vein, and the hepatic artery were the vessels repaired in the 17 cases included in the study, Portal Vein (PV) + Superior Mesenteric Artery (SMA) repair was done in one (5.88%) and hepatic artery in one (5.88%). Type I reconstruction was done in nine (52.94 %) (four (23.52%) for PV and five (29.41%) for SMV). Type III reconstruction was done in five (29.41%) (Three (17.64%) PV and two (11.76%) SMV resections). Type IV reconstruction was done in one (5.88%) patient for PV (Table 4).

**Table 4:** Type of vascular reconstruction (n= 17).

Vessel reconstructed	Type of reconstruction	n (%)	Remarks
Portal vein + Superior mesenteric artery (PV+SMA)	NA^*^	1 (5.88)	
Hepatic artery (HA)	NA	1 (5.88)	POPF^†^ and DGE^‡^
Portal vein (PV)	Type I	4 (23.5)	POPF in one
	Type II	-	
	Type III	3 (17.64)	
	Type IV	1 (5.88)	Mortality
Superior mesenteric vein (SMV)	Type I	5 (29.4)	
	Type II	-	
	Type III	2 (11.76)	GOO^§^, DGE in one
	Type IV	-	

* NA = not applicable

† POPF = Postoperative Pancreatic Fistula

‡ DGE = Delayed Gastric Emptying

§ GOO = Gastric Outlet Obstruction

Tolerance of oral liquid diet, time of appearance of bowel sound, time of passage of flatus/stool, vomiting, time of reinsertion of NG tube (if required), post-op days of IV fluid, tolerance of normal diet, and discharge were noted (Table 5).

**Table 5:** Postoperative outcomes (n= 17).

Outcomes	Median duration
Appearance of bowel sound	24-48 hours
Time of passage of flatus/stool	48-72 hours
IV fluid stopped	3 days
Discharge	7 days
	n (%)
Tolerance of oral liquids from 1^st^ POD^*^	15 (88.2)
Reinsertion of NG tube	2 (11.7)
Tolerance of normal diet	15 (88.2)
Readmission	3 (17.6)
Incidence of CrDGE^†^	2 (11.7)
Incidence of CrPOPF^‡^	2 (11.7)
Clavien-Dindo ≥3	1 (5.88)

* POD = Postoperative Day

† Cr-DGE = Clinically relevant Delayed Gastric Emptying

‡ Cr-POPF = Clinically relevant Postoperative Pancreatic Fistula

## DISCUSSION

In this study we looked for tolerance of early oral feeding with vascular resection in patients undergoing pancreatoduodenectomy. Naso gastric tube was avoided in all patients from the day of surgery and oral sips of clear liquid was started from the same day. Fifteen (88%) patients tolerated early oral feeding well. Two patients required some form of intervention during hospital stay. CrDGE and CrPOPF were noted in only two (11.7%) of patients. In a normal individual antral contractions are believed to be more important in emptying the solid bowel content. Pressure gradient between the stomach and the duodenum plays a role in gastric emptying. The common pressure gradient of the stomach and small bowel, after gastric accommodation following gastrojejunostomy, influences the emptying of liquids in patients with pancreatoduodenectomy.^7^

Factors that play a major role in oral feeding intolerance are the disruption of vagal innervation, the diminished function of the circular smooth muscle and interstitial cells of Cajal, loss of the antropyloric coordination, and hormonal influences.^8^ A more radical approach to surgery can accentuate such factors. Also, duodenal resection done in all pancreatoduodenectomy leads to reduced motilin secretion which contributes to the suppression of migrating motor complex thus contributing to gastric retention.^9^

The first vascular resection with reconstruction was reported by Moore GE, et al. as a part of pancreatoduodenectomy in the year 1951.^10^ The results of such surgery is also getting better and a similar overall survival is reported in patients undergoing venous resection to attain R_0_ when compared to those without venous resection.^11^ The en bloc vascular resection for SMV/PV has become standard practice in many centers, the ability to achieve R_0_ resection has been reported to be up to 98%.^12,13^ Morbidity in the postoperative period between the vascular resection and non vascular resection group is reported to be similar.^13^

To our knowledge there are no other studies done to see the tolerance of oral feeding and DGE alone in pancreatoduodenectomy with vascular resection. Age of the patient, BMI, preoperative cholangitis, and underlying diabetes mellitus are a few of the identified risk factors associated with a greater chance of oral feeding intolerance in pancreatoduodenectomy patients. Low preoperative albumin level is also associated with increased morbidity.^14^ In our series 41.1% patients had preoperative hypoalbuminemia.

One of the reasons for intolerance of early feeding could be the practice of pylorus preservation. In our practice, we resect the pylorus at the time of gastric resection (PPPD). The first PPPD related paper was published in 1978.^15^ The technique PPPD was proposed initially to preserve the physiological function of the gastrointestinal tract with its benefit on digestion and nutritional status and prevent postgastrectomy dumping syndrome.^16^ The denervation and devascularization of the pylorus result in pylorospasm so it contributes to the development of oral feeding intolerance.^17^ Warshaw AL, et al. were the first to associate DGE with PPPD.^18^ A systematic review published from Newcastle in 2021 stated that in 71 percent of comparisons PPPD appeared to be the best approach for decreasing DGE.^19^ Other studies have also shown similar reports.^20^

After gastric resection, continuity of the gastrointestinal tract is maintained by a gastrojejunostomy. The route of gastrointestinal reconstruction is done either antecolic (AC) or retrocolic (RC). The preferred choice of such reconstruction is a subject of discussion.^21^ In an RCT published in 2014 showed the two routes of reconstruction did not affect the incidence of postoperative DGE.^21^ Two other systematic reviews and meta-analyses on the issue showed the AC route to be better to decrease the incidence of DGE, thus leading to a better tolerance of early oral feeds, but these studies had the limitation of having a smaller sample size. A Chinese meta-analysis published in 2019, including many randomized controlled trials and retrospective comparative studies comparing the outcome of AC vs RC route of GJ, showed a significantly fewer incidence of DGE in the AC group.^22^ When only RCTs were compared separately in this study the rate of DGE in the AC group was still lesser by 0.71% (95% CI, 0.50-1.01; P= 0.05).^22^ In a separate evaluation of the retrospective comparative studies also the rate of DGE was significantly lesser by 0.35% (95% CI, 0.27-0.46; P<0.00001).^22^ A similar conclusion was published in the meta-analysis released in 2021 from Newcastle where the rate of DGE was reported to be higher in the RC group than in the AC group.^19^ Tight transmesocolic window could be a cause of less tolerance of early oral feeding leading to DGE in a patient undergoing RC reconstruction.^19^ In our practice, we use the AC route to perform gastrojejunostomy. This could be the reason why early feeding was well tolerated in our patients.

Postoperative pancreatic fistula (POPF) is a major determinant of early oral feeding. After resection of the pancreatic head, there are many techniques of pancreatico enteric anastomosis.^23,24^ In this series pancreaticojejunostomy was performed by the Blumgart duct-to-mucosa transpancreatic U suturing technique. In a study comparing the rate of CrPOPF between the Blumgart anastomosis technique vs others, the Blumgart technique was reported to have a decrease in the rate of formation of CrPOPF (0.67-7.14 %); which is significantly less than the other methods (10-20%).^25^ By the use of the trans pancreatic U suture, the Blumgart anastomosis technique reduces the shearing force at the pancreatic stump. By the use of the interrupted mattress U-sutures, the blood supply to the pancreatic stump is adequate. The technique also guarantees excellent visualization of the pancreatic duct while placing duct-to-mucosal sutures, as it is placed in the beginning. It ensures tension-free approximation between the posterior and anterior seromuscular layer of the jejunum with the pancreatic capsule.^41^ Lesser frequency of CrPOPF (11.7%) due to the application of this technique can be attributed to a lesser degree of DGE in our patients and thus a better tolerance of early oral feeding.^26^

A publication from the Netherlands in the year 2013 studied the association of preoperative symptoms of gastric outlet obstruction (GOO) with feeding intolerance after pancreatoduodenectomy. It included the preoperative symptoms of nausea, vomiting, loss of appetite, postprandial discomfort, dysphagia, and weight loss as the symptoms of GOO. Except for weight loss, the presence of more than two symptoms of GOO was recognized as the independent recognizable risk factor for developing DGE in the post-operative period.^27^

Although these symptoms were not separately studied in our study, the mode of presentation of the patients was noted. In our patients only 2 (11.7%) of the patient had preoperative symptoms suggestive of GOO. The lesser no of DGE in our series can be justified due to this reason as well.

In our practice, we initiate the use of prokinetic agents from day 1 of surgery. Metoclopramide, domperidone, and erythromycin are the commonly used agents for such purposes. Erythromycin (a motilin agonist) combined with motilin receptor enhances gastric emptying.^28^ Erythromycin has been used for a long time for the reduction of DGE. RCTs have highlighted the role of erythromycin to prevent DGE in the past.^29^ Routine use of such prokinetic agents has helped reduce the incidence of feeding intolerance in our series.

The cases subjected for vascular resection are mostly advanced cases. Extensive lymph node dissection along the whole of mesopancreas is done while operating on these patients. Triangle surgery was also performed in some of the cases (n= 5) in our series. This has resulted in the complete removal of the celiac plexus during lymphadenectomy which results in complete sympathetic denervation and unopposed parasympathetic stimulation of the GI tract.^30^ Oral feeding is well tolerated in these patients as the unopposed parasympathetic stimulation helps in gastric emptying.

So our study shows that when all these factors and techniques are implemented, early oral feeding is tolerated well by the patients. Since it's a descriptive cross sectional study with small sample size done in single center, its results cannot be generalized as of now. A well-designed prospective study with a larger sample size is advocated.

## CONCLUSIONS

The prevalence of early oral feeding with vascular resection among patients undergoing pancreatoduodenectomy was similar to other studies done in similar setting. Various factors play its part to help the patients tolerate oral feeding and avoid the chances of developing delayed gastric emptying. Early oral feeding is well tolerated recommended for patients undergoing pancreatoduodenectomy with vascular resection.

## References

[1] Muscaritoli M, Arends J, Bachmann P, Baracos V, Barthelemy N, Bertz H (2021). ESPEN practical guideline: Clinical Nutrition in cancer.. Clin Nutr..

[2] Thapa PB, Nagarkoti K, Lama T, Maharjan DK, Tuladhar M (2011). Early enteral feeding in intestinal anastomosis.. J Nepal Health Res Counc..

[3] Lassen K, Coolsen MME, Slim K, Carli F, De Aguilar-Nascimento JE, Schafer M (2013). Guidelines for perioperative care for pancreaticoduodenectomy: Enhanced recovery after surgery (ERAS®) society recommendations.. World J Surg..

[4] Matsunaga H, Tanaka M, Naritomi G, Yokohata K, Yamaguchi K, Chijiiwa K (1998). Effect of leucine 13-motilin (KW5139) on early gastric stasis after pylorus-preserving pancreatoduodenectomy.. Ann Surg..

[5] Sugiyama M, Abe N, Ueki H, Masaki T, Mori T, Atomi Y (2004). A new reconstruction method for preventing delayed gastric emptying after pylorus-preserving pancreatoduodenectomy.. Am J Surg..

[6] Bassi C, Marchegiani G, Dervenis C, Sarr M, Abu Hilal M, Adham M (2017). The 2016 update of the International Study Group (ISGPS) definition and grading of postoperative pancreatic fistula: 11 Years After.. Surgery..

[7] Indireshkumar K, Brasseur JG, Faas H, Hebbard GS, Kunz P, Dent J (2000). Relative contributions of "pressure pump" and "peristaltic pump" to gastric emptying.. Am J Physiol Gastrointest Liver Physiol..

[8] Goyal RK, Guo Y, Mashimo H (2019). Advances in the physiology of gastric emptying.. Neurogastroenterol Motil..

[9] Deloose E, Janssen P, Depoortere I, Tack J (2012). The migrating motor complex: control mechanisms and its role in health and disease.. Nat Rev Gastroenterol Hepatol..

[10] Moore GE, Sako Y, Thomas LB (1951). Radical pancreatoduodenectomy with resection and reanastomosis of the superior mesenteric vein.. Surgery..

[11] Ravikumar R, Sabin C, Abu Hilal M, Bramhall S, White S, Wigmore S (2014). Portal vein resection in borderline resectable pancreatic cancer: a United Kingdom multicenter study.. J Am Coll Surg..

[12] Tempero MA, Malafa MP, Al-Hawary M, Asbun H, Bain A, Behrman SW (2017). Pancreatic Adenocarcinoma, Version 2.2017, NCCN Clinical Practice Guidelines in Oncology.. J Natl Compr Canc Netw..

[13] Worni M, Castleberry AW, Clary BM, Gloor B, Carvalho E, Jacobs DO (2013). Concomitant vascular reconstruction during pancreatectomy for malignant disease: a propensity score-adjusted, population-based trend analysis involving 10,206 patients.. JAMA Surg..

[14] Akizuki E, Kimura Y, Nobuoka T, Imamura M, Nagayama M, Sonoda T (2009). Reconsideration of postoperative oral intake tolerance after pancreaticoduodenectomy: prospective consecutive analysis of delayed gastric emptying according to the ISGPS definition and the amount of dietary intake.. Ann Surg..

[15] Traverso LW, Longmire WP (1978). Preservation of the pylorus in pancreaticoduodenectomy.. Surg Gynecol Obstet..

[16] Itani KM, Coleman RE, Meyers WC, Akwari OE (1986). Pylorus-preserving pancreatoduodenectomy. A clinical and physiologic appraisal.. Ann Surg..

[17] Kim DK, Hindenburg AA, Sharma SK, Suk CH, Gress FG, Staszewski H (2005). Is pylorospasm a cause of delayed gastric emptying after pylorus-preserving pancreaticoduodenectomy?. Ann Surg Oncol..

[18] Warshaw AL, Torchiana DL (1985). Delayed gastric emptying after pylorus-preserving pancreaticoduodenectomy.. Surg Gynecol Obstet..

[19] Varghese C, Bhat S, Wang TH, O'Grady G, Pandanaboyana S (2021). Impact of gastric resection and enteric anastomotic configuration on delayed gastric emptying after pancreaticoduodenectomy: a network meta-analysis of randomized trials.. BJS Open.

[20] Buchler MW, Friess H, Muller MW, Wheatley AM, Beger HG (1995). Randomized trial of duodenum-preserving pancreatic head resection versus pylorus-preserving Whipple in chronic pancreatitis.. Am J Surg..

[21] Eshuis WJ, van Eijck CH, Gerhards MF, Coene PP, de Hingh IH, Karsten TM (2014). Antecolic versus retrocolic route of the gastroenteric anastomosis after pancreatoduodenectomy: a randomized controlled trial.. Ann Surg..

[22] Qiu J, Li M, Du C (2019). Antecolic reconstruction is associated with a lower incidence of delayed gastric emptying compared to retrocolic technique after Whipple or pylorus-preserving pancreaticoduodenectomy.. Medicine (Baltimore)..

[23] Thapa PB, Maharjan DK, Regmi S (2017). Pancreatic anastomosis: challenges and outcomes.. J Surg Transplant Sci..

[24] Kakita A, Takahashi T, Yoshida M, Furuta K (1996). A simpler and more reliable technique of pancreatojejunal anastomosis.. Surg Today..

[25] Li Z, Wei A, Xia N, Zheng L, Yang D, Ye J (2020). Blumgart anastomosis reduces the incidence of pancreatic fistula after pancreaticoduodenectomy: a systematic review and meta-analysis.. Sci Rep..

[26] Wang X, Bai Y, Cui M, Zhang Q, Zhang W, Fang F (2018). Modified Blumgart anastomosis without pancreatic duct-to-jejunum mucosa anastomosis for pancreatoduodenectomy: a feasible and safe novel technique.. Cancer Biol Med..

[27] Atema JJ, Eshuis WJ, Busch OR, van Gulik TM, Gouma DJ (2013). Association of preoperative symptoms of gastric outlet obstruction with delayed gastric emptying after pancreatoduodenectomy.. Surgery..

[28] Ohwada S, Satoh Y, Kawate S, Yamada T, Kawamura O, Koyama T (2001). Low-dose erythromycin reduces delayed gastric emptying and improves gastric motility after Billroth I pylorus-preserving pancreaticoduodenectomy.. Ann Surg..

[29] Yeo CJ, Barry MK, Sauter PK, Sostre S, Lillemoe KD, Pitt HA (1993). Erythromycin accelerates gastric emptying after pancreaticoduodenectomy. A prospective, randomized, placebo-controlled trial.. Ann Surg..

[30] Erdine S (2005). Celiac ganglion block.. Agri..

